# Comparative analysis of different hepatico-jejunostomy techniques for treating adult type I choledochal cyst

**DOI:** 10.1093/gastro/gox025

**Published:** 2017-07-02

**Authors:** Wenjie Ma, Yongqiong Tan, Anuj Shrestha, Fuyu Li, Rongxing Zhou, Junke Wang, Haijie Hu, Qin Yang

**Affiliations:** 1 Department of Biliary Surgery, West China Hospital of Sichuan University, Chengdu, China; 2 Operation Room, West China Hospital of Sichuan University, Chengdu, China; 3 Department of General Surgery, Andaki Medical College, Pokhara, Nepal

**Keywords:** Choledochal cyst, roux-en-Y hepatico-jejunostomy, biliary drainage, adults

## Abstract

**Objective:**

To compare Roux-en-Y hepatico-jejunostomy with complete resection of the cyst or incomplete resection with 1-cm remnant proximal cyst wall in treating adult type I choledochal cyst (CC).

**Methods:**

The medical records of 267 adult patients with type I CC from January 1998 to December 2015 were reviewed retrospectively. Among them, 171 underwent Roux-en-Y hepatico-jejunostomy with complete resection (PBD 0-cm group) and 96 underwent Roux-en-Y hepatico-jejunostomy with 1-cm proximal cyst wall left (PBD 1-cm group). The short- and long-term post-operative complications were compared between the two groups.

**Results:**

No significant difference was observed in operative time or anastomotic diameter between the two groups. The incidence of perioperative complications was significantly higher in the PBD 1-cm group than that in the PBD 0-cm group (28.1% vs 14.0%, *p*=0.005), especially post-operative cholangitis (7.3% vs 1.2%, *p*=0.021). The incidence of long-term post-operative complications was not significantly different, including anastomotic stricture, reflux cholangitis, intra-hepatic bile duct stones and bile leak (all *p *>0.05). Post-operative intra-pancreatic biliary malignancy occurred in one patient in the PBD 0-cm group at 25 months and one patient in the PBD 1-cm group at 5 month, respectively. Anatomical site malignancy was observed in one patient in the PBD 1-cm group at 10 months.

**Conclusion:**

Ease of performing anastomosis does not justify retaining a segment of choledochal cyst in type I CC due to its higher risk of post-operative complication and malignancy. A complete excision of the CC with anastomosis to the healthy proximal bile duct is necessary in treatment of type I CC.

## Introduction

Type I choledochal cyst (CC) is a rare congenital cystic dilation of the extra-hepatic biliary tract, but its exact etiology is not clear [1–3]. With advances in medical technology, the number of CC diagnoses in adult patients has been increasing, with more than 65.7% of the total adult patients [4]. Type I CC is associated with complications such as cholangitis, pancreatitis, cholelithiasis, spontaneous rupture and malignant transformation. An increase in the risk of malignancy with age has been documented in the literature. The reported incidence varies from 2.5% to 28%, with up to 50% in patients over 50 years old [1–3,5–7]. Most CCs are associated with anomalous pancreaticobiliary duct union, which also causes carcinogenesis of the biliary tree by reflux of pancreatic juice into the bile duct [8–10]. Further, it was found that cancers could develop in any remnant cyst including the remaining hilar bifurcation, intra-hepatic or intra-pancreatic cysts. Therefore, some scholars believe that CC should be completely resected, including the involved hilar bifurcation and intra-pancreatic segment. Total resection of the CC along with Roux-en-Y hepatico-jejunostomy (RYHJ) is the most widely accepted strategy because of the simplicity of the procedure and lower risk of post-operative malignancy [2,3,11].

To decrease the complexity of the reconstruction in difficult situations, and to decrease the risk of anastomotic stricture, according to some reports, 0.5–1.0 cm of the proximal cyst walls could be left behind to facilitate biliary anastomosis [3,6,12]. However, it has been reported that the incidence of anastomotic stricture is not higher in patients who undergo complete resection compared to those with a part of the cyst wall remained [2,13,14]. Further, the incidence of long-term post-operative anastomotic malignant transformation after the proximal cystic wall remnant is left behind is 0.7–5.4%, which is higher than that in the general population [9,13,15]. Incomplete resection of CC leads to an increase in the carcinogenesis rate and the major cause of cancer development was the remnant of the distal choledochal cyst or bile duct [13,16].

In this study, to investigate whether the 1-cm left proximal cyst wall decreases the risk of post-operative complications including anastomotic stricture, reflux cholangitis and malignancy, we compared the effects of complete resection of the proximal cyst with incomplete resection in which a 1-cm area of the proximal CC wall is left behind.

## Patients and methods

### Patients selection

A retrospective analysis of a database of 267 adult patients (≥18 years old) diagnosed with type I CC between January 1998 and December 2015 was conducted in our center. All the patients were diagnosed with type I CC based on ultrasonography, and computerized tomography (CT) or magnetic resonance cholangiopancreatography (MRCP). Further, the diagnosis was confirmed in all the cases by histopathological examination of the resected specimens. The patients were divided into two groups: (i) those who underwent complete resection of the involved hepatic bifurcation plus RYHJ (proximal bile duct 0-cm left, PBD 0-cm group); and (ii) those who underwent incomplete resection with 1 cm of the proximal CC wall left behind to facilitate RYHJ (proximal bile duct 1-cm left, PBD 1-cm group). Surgical methods were selected based on whether the hepatic bifurcation was involved by cyst and the diameters of the left and right hepatic ducts.

### Surgical procedures

After careful dissection and division of the cyst, we opened the cyst wall and identified the luminal appearance grossly to determine the proximal transection line. The diameter of the proximal cutting edge, left and right hepatic duct was checked. In cases of insufficient diameter from the bile duct stump(s), the preparation was continued cranially, until satisfactory diameter is achieved.

**Figure 1:**
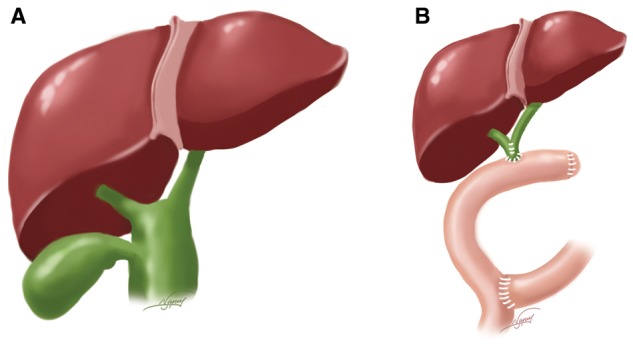
(**A**) If the diameter of the left and right hepatic ducts is > 1 cm and the hepatic bifurcation was involved by cysts intra-operatively, (**B**) left and right hepatic ducts were transformed into a common channel using 4–0 absorbed sutures interrupted stitches to perform Roux-en-Y hepatico-jejunostomy.

**Figure 2:**
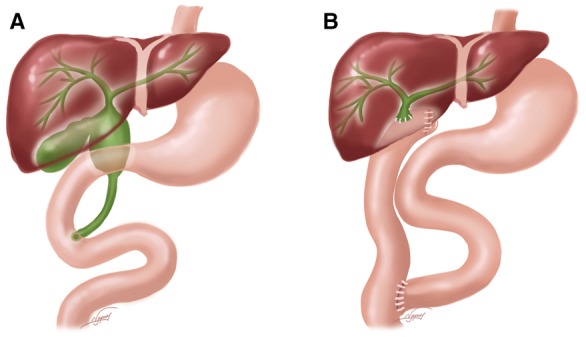
(**A**) If the diameter of the left and right hepatic ducts was ≤1 cm and the cysts were not involved in the hepatic bifurcation, (**B**) 1-cm proximal choledochal cyst wall would be left for Roux-en-Y hepatico-jejunostomy.

When the diameter of the left and right hepatic ducts was >1 cm and the hepatic bifurcation is involved by the cyst, completely resecting the proximal cyst and the involved hepatic bifurcation, left and right hepatic ducts were transformed into a common channel for a wider hepatico-jejunostomy, using 4–0 absorbed sutures interrupted stitches (**Figure 1**). If the diameter of the left and right hepatic ducts is ≤1 cm and the cyst is not involved the hepatic bifurcation, or the diameter of the proximal cutting edge would be <1.5 cm after resecting hepatic bifurcation according to an experienced surgeon’s evaluation, 1-cm proximal CC wall would be left for RYHJ to get a wider hepatico-jejunostomy (**Figure 2**).

For cases whose hepatic bifurcation was resected, the anastomotic diameter was measured intra-operatively. If the diameter of the anastomosis is <1.5 cm, we prefer to place a transanastomotic T-tube/orthopedic stent in order to protect and improve the patency of the anastomosis in the post-operative period [17,18]. We usually use a 16 Fr T-tube in case of small bile ducts. The RYHJ was performed in the same way as the Demetrios technique [18]. The parameters assessed included the demographic data, presenting symptoms, perioperative findings, post-operative complications and long-term outcomes. Patients with concurrent advanced biliary cancer were not included in this study. The study protocol was approved by the Institutional Review Board of West China Hospital, and the need for patient consent was waived because of the retrospective design. Written informed consent was obtained from each patient for the surgical procedures performed.

### Follow-up

All the patients were followed up by the same team of surgeons after the surgery. The patients were regularly followed up via outpatient check-ups and telephone. Physical examinations, abdominal ultrasonographic studies and biochemical examinations were conducted at each visit. Additional examinations, including CT and/or MRCP, were conducted to check for cholangitis, stenosis, stones or even advanced tumors. Patients with stenosis or choledocholithiasis were treated conservatively if they had no symptoms or mild symptoms. If a patient had severe stenosis, choledocholithiasis or a life-threatening episode of acute cholangitis, or if the patient did not respond to initial conservative treatment, a definitive surgical procedure was considered. Radical resection was suggested for patients with cholangiocarcinoma.

### Statistical analysis

Quantitative data that were normally distributed were reported as mean ± standard deviation and those that were not normally distributed were presented with the median (range) values. Statistical analyses were carried out using SPSS 24.0 (IBM SPSS Statistics, IBM Corp., Armonk, NY). All relevant pre-operative, intra-operative and post-operative factors were recorded. Qualitative data were analysed using the chi-square test and Fisher’s exact test. Quantitative data were analysed using *t*-tests and those that were not normally distributed were analysed using the rank-sum test. Statistical significance was set at *p *< 0.05.

To correct for differences in covariates that could affect choosing the method of type I CC surgery, and to reduce the selection bias inherent in retrospective observational studies, a propensity score matching analysis was performed between the PBD 0-cm and 1-cm groups. To estimate the propensity score, a function was built using a logistic regression model for the method of surgery on the basis of the patient’s clinical factors. The propensity score was calculated with pre-operative factors included age, sex, American Society of Anesthesiologist physical status classification (ASA score) and diameter of the left and right hepatic duct.

## Results

This study included 267 patients. The median follow-up period after surgery was 66 months (range, 21–216 months). The PBD 0-cm group comprised 171 patients and the PBD 1-cm group included 96 patients. The female:male ratios in the 0-cm and 1-cm groups were 3.275 and 2.840, respectively. The median age of the 0-cm group was 34 years (range, 18–68 years), which did not significantly differ from the median age of 35 years (range, 18–70 years) in the 1-cm group (*p*=0.13). Seven patients (4.1%) in the PBD 0-cm group and seven patients (5.2%) in the PBD 1-cm group had an ASA score of 3 or more (*p*=0.26). The most common symptom was intermittent abdominal pain, which was reported in 155 (90.6%) patients in the PBD 0-cm group and 74 (77.1%) patients in the PBD 1-cm group, respectively (*p*=0.002). Coexistent hepatobiliary-pancreatic (HBP) disease was found in 50.2% patients. There is no significant difference between these two groups for HBP-associated diseases (48.5% vs 53.1%, *p*=0.47). The pre-operative data are summarized in **Table 1**.

**Table 1:** Summary of pre-operative data

Variable	Total (*n*=267)	PBD 0-cm group (*n*=171)	PBD 1-cm group (*n*=96)	*P-*value
Median age, years	35 (18–70)	34 (18–68)	35 (18–70)	0.13
Female, *n* (%)	205 (76.8)	131 (76.6)	71 (74.0)	0.63
ASA score ≥3, *n* (%)	14 (5.2)	7 (4.1)	7 (7.3)	0.26
**Clinical presentation**				
Asymptomatic, *n* (%)	17 (6.4)	9 (5.3)	8 (8.3)	0.32
Symptomatic, *n* (%)	250 (93.6)	162 (94.7)	88 (91.7)	0.32
Abdominal pain	229 (85.8)	155 (90.6)	74 (77.1)	0.002
Jaundice	58 (21.7)	43 (25.1)	15 (15.6)	0.07
Abdominal mass	8 (3.0)	7 (4.1%)	1 (1.0%)	0.30
Classic triad	4 (1.5)	4 (2.3)	0 (0.0)	0.33
Median delay between symptom and diagnosis, months	12 (0.0–576)	12 (0.1–576)	11 (0.0–360)	0.92
**Associated hepatobiliary-pancreatic disease**				
Total, *n* (%)	134 (50.2)	83 (48.5)	51 (53.1)	0.47
Hepatic, *n* (%)	2 (0.75)	2 (1.2)	0 (0.0)	1.00
Hepatic abscess	2 (0.75)	2 (1.2)	0 (0.0)	1.00
Biliary, *n* (%)	134 (50.2)	83 (48.5)	51 (53.1)	0.47
Gallstones/cholecystitis	48 (18.0)	33 (19.3)	15 (15.6)	0.45
Cholangitis	34 (12.7)	16 (9.4)	18 (18.4)	0.027
Biliary lithiasis	96 (36.0)	61 (35.7)	35 (36.5)	0.90
Intra-hepatic stones	4 (1.5)	3 (1.8)	1 (1.0)	1.00
Choledocolithiasis	92 (34.5)	58 (33.9)	34 (35.5)	0.81
Pancreatic, *n* (%)	9 (3.4)	8 (4.7)	1 (1.0)	0.22
Pancreatitis	9 (3.4%)	8 (4.7%)	1 (1.0)	0.22
Pancreatic calculi formation	3 (1.1)	3 (1.8)	0 (0.0)	0.48
Median diameter of cyst, cm	3 (0.5–17)	3 (1.7–13)	3 (2.1–17)	0.59

A summary of the surgical treatments is shown in **Table 2**. Resection of the cyst and RYHJ was completed in all 267 patients. The operative time and intra-operative blood loss were not significantly different between the two groups. The median diameters of the left and right hepatic duct in the PBD 0-cm group were significantly higher than those in the PBD 1-cm group [left: 1.5 (0.8–4.0) cm vs 1.1 (0.3–3.7) cm, *p*=0.04; right: 1.5 (0.8–4.0) cm vs 1.2 (0.3–3.7) cm, *p*=0.03]. However, no significant difference was observed with regard to the anastomotic diameter [1.5 (0.8–3.0) cm vs 1.5 (0.7–3.0) cm; *p*=0.58]. The intra-operative placement rate of the anastomotic support tube (T-tube or orthopedic drainage tube) in the PBD 0-cm group was higher than that in the PBD 1-cm group (2.9% vs 1.0%), but the difference was not statistically significant (*p*=0.57).

**Table 2:** Patient characteristics and perioperative findings: unadjusted and propensity score (PS) matched between the PBD 0-cm group and the PBD 1-cm group

Variable	Before PS matching			After PS matching		
	PBD 0-cm group (*n*=171)	PBD 1-cm group (*n*=96)	*P-*value	PBD 0-cm group (*n*=86)	PBD 1-cm group (*n*=96)	*P-*value
**Demographics**						
Median age, years	34 (18–68)	35 (18–70)	0.13	34 (15–64)	35 (18–70)	0.14
Female, *n* (%)	131 (76.6)	71 (74.0)	0.63	26/60	25/71	0.53
ASA score ≥3, *n* (%)	7 (4.1)	7 (7.3)	0.26	5 (5.8)	7 (7.3)	0.69
Symptom, *n* (%)	162 (94.7)	88 (91.7)	0.32	83 (96.5)	88 (91.7)	0.17
Associated hepatobiliary-pancreatic disease, *n* (%)	80 (46.8)	51 (53.1)	0.32	40 (46.5)	51 (53.1)	0.37
**Perioperative finding**						
Median operative time, min	225 (110–760)	225 (105–505)	0.64	226.5 (120–760)	225 (105–505)	0.93
Median estimated blood loss, ml	150 (30–2000)	150 (50–1000)	0.24	200 (50–2000)	150 (50–1000)	0.095
Median diameter of left hepatic duct, cm	1.5 (0.8–4.0)	1.1 (0.3–3.7)	0.04	1.2 (0.5–3.3)	1.2 (0.3–4.0)	0.41
Median diameter of right hepatic duct, cm	1.5 (0.8–4.0)	1.2 (0.3–3.7)	0.03	1.1 (0.5–3.3)	1.1 (0.3–4.0)	0.48
Median anastomotic diameter, cm	1.5 (0.8–3.0)	1.5 (0.7–3.0)	0.58	1.5 (1.0–3.0)	1.5 (0.7–3.0)	0.98
Biliary stent, *n* (%)	5 (2.9)	1 (1.0)	0.57	3 (3.5)	1 (1.0)	0.54
Post-operative hospital stay, days	8 (5–40)	8 (4–69)	0.35	8 (5–40)	8 (4–69)	0.66

### Perioperative complications

The overall perioperative mortality rate was 1.5% (4/267) and the perioperative morbidities are shown in **Table 3**. The overall perioperative morbidity rate in the PBD 0-cm group was 14.0%, including cholangitis (*n*=2), intra-abdominal infection (*n*=8), pancreatitis (*n*=6), bile leak (*n*=11), pancreatic leak (*n*=7), wound infection (*n*=12), hepatocerebral disease (*n*=1) and intra-abdominal bleeding (*n*=5). The overall perioperative morbidity rate in the PBD 1-cm group was 28.1%, including cholangitis (*n*=7), pancreatitis (*n*=5), bile leak (*n*=5), pancreatic leak (*n*=3), wound infection (*n*=4), intra-abdominal infection (*n*=3), pulmonary infection (*n*=2) and intra-abdominal bleeding (*n*=4). The overall complication rate was significantly higher in the PBD 1-cm group (28.1% vs 14.0%, *p*=0.005), especially post-operative cholangitis (7.3% vs 1.2%, *p*=0.021). According to the Clavien-Dindo classification of surgical complications [19], severe complications (grade ≥III) occurred in five (3.3%) patients in the PBD 0-cm group and in three (3.1%) patients in the PBD 1-cm group.

**Table 3:** Perioperative complication: unadjusted and propensity score (PS) matched between the PBD 0-cm group and the PBD 1-cm group

Perioperative complication	Before PS matching			After PS matching		
	PBD 0-cm group (*n*=171)	PBD 1-cm group (*n*=96)	*P-*value	PBD 0-cm group (*n*=86)	PBD 1-cm group (*n*=96)	*P-*value
Total, *n* (%)	24 (14.0)	27 (28.1)	0.005	9 (10.5)	27 (28.1)	0.003
Cholangitis, *n* (%)	2 (1.2)	7 (7.3)	0.021	2 (2.3)	7 (7.3)	0.23
Pancreatitis, *n* (%)	6 (3.5)	5 (5.2)	0.73	2 (2.3)	5 (5.2)	0.53
Bile leak, *n* (%)	11 (6.4)	5 (5.2)	0.69	6 (7.0)	5 (5.2)	0.85
Duration of bile leak, days	5 (3–7)	5 (3–12)	0.82	5 (3–7)	5 (3–12)	0.85
Pancreatic juice leak, *n* (%)	7 (4.1)	3 (3.1)	0.95	1 (1.2)	3 (3.1)	0.69
Duration of pancreatic leak, days	4 (2–22)	4 (3–7)	0.73	2	4 (3–7)	0.18
Surgical site infection, *n* (%)	12 (7.0)	4 (4.2)	0.35	4 (4.7)	4 (4.2)	1.00
Intra-abdominal infection, *n* (%)	8 (4.7)	3 (3.1)	0.77	1 (1.2)	3 (3.1)	0.69
Pulmonary infection, *n* (%)	0 (0.0)	2 (2.1)	0.25	0 (0.0)	2 (2.1)	0.53
Hepatocerebral disease, *n* (%)	1 (0.6)	0 (0.0)	1.00	1 (1.2)	0 (0.0)	0.96
Intra-abdominal bleeding, *n* (%)	5 (3.3)	4 (4.2)	0.85	3 (3.5%)	4 (4.2)	1.00
Reintervention, *n* (%)	6 (3.5)	3 (3.1)	0.73	2 (2.3)	3 (3.1)	1.00
Mortality, *n* (%)	2 (1.2)	2 (2.1)	0.95	2 (2.3)	2 (2.1)	1.00

### Long-term complications

During the 21–216 months’ follow-up, 32 patients (18.7%) in the PBD 0-cm group suffered from long-term complications and the proportion was slightly lower than the PBD 1-cm group (21.9%, *p*=0.53) (**Table 4**). During the follow-up period, the rate of anastomotic stricture (5.8% vs 7.3%), reflux cholangitis (10.5% vs 14.6%), post-operative pancreatitis (0.6% vs 2.1%), liver abscess (0.6% vs 4.2%) and biliary calculi formation (8.2% vs 10.4%) in the PBD 0-cm group was slightly lower than that in the PBD 1-cm group; and delayed biliary leaks (4.1% vs 2.1%) in the PBD 0-cm group were higher than those in the PBD 1-cm group. However, these differences were not statistically significant (all *p *> 0.05). In the PBD 0-cm and 1-cm groups, one patient was found to have unresectable malignant transformation in the remnant distal stump at 25 months and at 5 months after surgery, respectively; and, in the PBD 1-cm group, one patient was found to have anastomosis site malignancy by CT 10 months after surgery.

**Table 4:** Long-term post-operative complication: unadjusted and propensity score (PS) matched between the PBD 0-cm group and the PBD 1-cm group

Long-term complications	Before PS matching			After PS matching		
	PBD 0-cm group (*n*=171)	PBD 1-cm group (*n*=96)	*P-*value	PBD 0-cm group (*n*=86)	PBD 1-cm group (*n*=96)	*P-*value
Total, *n* (%)	32 (18.7)	21 (21.9)	0.53	16 (18.6)	21 (21.9)	0.58
Anastomotic stenosis, *n* (%)	10 (5.8)	7 (7.3)	0.64	2 (2.3)	7 (7.3)	0.23
Adhesive intestinal obstruction, *n* (%)	3 (1.8)	1 (1.0)	1.00	2 (2.3)	1 (1.0)	0.92
Reflux cholangitis, *n* (%)	18 (10.5)	14 (14.6)	0.33	8 (9.3)	14 (14.6)	0.28
Reflux cholangitis, times/year	3 (1∼24)	3 (1∼24)	0.72	3 (1∼24)	3 (1∼24)	0.87
Pancreatitis, *n* (%)	1 (0.6)	2 (2.1)	0.61	0 (0.0)	2 (2.1)	0.53
Hepatic abscess, *n* (%)	1 (0.6)	4 (4.2)	0.11	1 (1.2%)	4 (4.2)	0.43
Calculi formation, *n* (%)	14 (8.2)	10 (10.4)	0.54	8 (9.3%)	10 (10.4)	0.80
Intra-hepatic	8 (4.7)	4 (4.2)	1.00	4 (4.7)	4 (4.2)	1.00
Extra-hepatic	5 (2.9)	5 (5.2)	0.54	4 (4.7)	5 (5.2)	1.00
Intra- and extra-hepatic	1 (0.6)	1 (1.0)	1.00	0 (0.0)	1 (1.0)	1.00
Hepatic cirrhosis, *n* (%)	1 (0.6)	1 (1.0)	1.00	1 (1.2)	1 (1.0)	1.00
Bile leak, *n* (%)	7 (4.1)	2 (2.1)	0.60	1 (1.2)	2 (2.1)	1.00
Fistula of abdominal wall formation, *n* (%)	2 (1.2)	0 (0.0)	0.75	1 (1.2)	0 (0.0)	0.96
Canceration, *n* (%)	1 (0.6)	2 (2.1)	0.61	1 (1.2)	2 (2.1)	1.00
Biliodigestive anastomosis, *n* (%)	0 (0.0)	1 (1.0)	0.77	0 (0.0)	1 (1.0)	1.00
Remnant distal stump, *n* (%)	1 (0.6)	1 (1.0)	1.00	1 (1.2)	1 (1.0)	1.00

### Propensity score (PS) matched analysis

After PS matching, the median age and cases associated with HBP disease were not significantly different between two groups (**Table 2**). Perioperative findings such as operative time, estimated blood loss and diameter of hepatic duct were not significantly different between the two groups, similarly to data before PS matching except for the diameter of the left and right hepatic ducts (**Table 2**). After matching, the perioperative complication rate in the PBD 0-cm group was still significantly higher than that in PBD 1-cm group (28.1% vs 10.5%, *p*=0.003; **Table 3**). The long-term post-operative complication rates in the PBD 0-cm group and PBD-1 cm group were still not significantly different (18.6% vs 21.9%, *p*=0.58; **Table 4**), similarly to those before PS matching.

## Discussion

The findings of our retrospective comparative analysis show that, although there is no significant difference in the long-term complications between the two approaches when performing resection of type I CC, the perioperative complication rate is significantly higher in the PBD 1-cm group. The remnant proximal cyst wall did not significantly decrease the risk of post-operative anastomotic stricture, reflux cholangitis or malignancy transformation.

According to the literature, suture technique, bile duct diameter, hypoalbuminemia (albumin <35 g/L) and blood supply to the bile duct seem to be important factors related to the occurrence of bile leak [20,21]. In our series, however, the incidences of bile leakage in the PBD 1-cm group did not significantly decrease. It seemed that hepaticoenterostomy with remnant proximal cyst wall did not decrease the risk of post-operative bile leakage, which has emerged as one of the most important factors in the causation of the biliary stricture [22].

Some scholars suggested that some of the proximal cyst walls could be left behind to facilitate biliary anastomosis in order to reduce the risk of anastomotic stricture, as anastomotic stricture can lead to reflux cholangitis, hepatolithiasis and even carcinoma in the intra-hepatic ducts [3,6,12]. However, few long-term outcomes of remnant proximal cysts on biliary anastomosis have been reported. Accurate recognition of the origin and termination of the cyst is the basis of complete cyst excision. It is clearly difficult to define the ends of cysts extending from the confluence of the hepatic duct to the junction of the common duct and pancreatic duct. It is also difficult to differentiate the normal bile duct endothelium from the cyst lining by intra-operative frozen section. In our procedure, we opened the cyst wall and identified the luminal appearance grossly to determine the proximal transection line. Besides that, whether left and right hepatic ducts are involved and their diameters should also be evaluated. Complete removal of hepatic bifurcation and the cyst should be performed if the diameters of the left and right hepatic ducts are >1 cm and/or the anastomotic diameters are >2 cm after excision of the hepatic bifurcation. If the diameters of the left and right hepatics are small and hepatic bifurcation does not involve cysts, proximal 1-cm cyst walls will be left to facilitate biliary anastomosis and reduce the risk of intractable anastomotic stricture. This is consistent with our findings that the diameters of the left and right hepatic ducts in the PBD 0-cm group were both significantly higher than those in the PBD 1-cm group before PS matching. As a result, bilioplasty combined with hepatico-jejunostomy after complete resection of hepatic bifurcation was chosen in the PBD 0-cm group. For relative smaller anastomosis, a continuous 6–0 or 7–0 absorb suture for the posterior layer and interrupted proline suture for the anterior layer would be used. For larger anastomosis, continuous or interrupted absorbable suture would be used, and all knots would be located outside the anastomosis. In the present study, even the anastomotic diameters in both two groups were similar [1.5 (0.8–3.0) cm vs 1.5 (0.7–3.0) cm, *p *> 0.05]. A few more patients in the PBD 0-cm group underwent intra-operative placement of the anastomotic support tube (T-tube or orthopedic drainage tube) (2.9% vs 1.0%, *p *> 0.05). It is possible that the anastomotic diameter in some patients after complete resection of the cyst was less than 2 cm, and that the anastomotic support tube was placed in order to reduce the long-term risk of anastomotic stricture [23]. During the post-operative follow-up, the rate of anastomotic strictures in the PBD 0-cm group seemed to be lower than that in the PBD 1-cm group, even after PS matching, which suggested that there were no significant benefits of leaving behind part of the proximal cyst wall when performing resection of type I CC. The occurrence of anastomotic stricture is related to the size of the anastomosis, blood supply to the anastomosis, the grade of inflammation, anastomotic technique (intermittent or continuous suture), type of thread used (absorbable suture or proline) and other factors except if the proximal cyst wall was left. Therefore, leaving behind a part of the cyst wall to facilitate an easier anastomosis neither has a sound basis nor is it supported by long-term results.

Besides anastomotic stricture, the other intractable complications included reflux cholangitis and hepatolithiasis. Recurrent episodes of reflux cholangitis not only cause great pain to the patients and increase the cost of follow-up care, but also increase the risk of stone formation and even malignant transformation. The Oddi’s sphincter is an important structure for maintaining normal bile duct pressure, regulating the secretion of bile and pancreatic juice, and preventing intestinal reflux. Biliary enteric anastomosis lacks the valve effect of the Oddi’s sphincter, which leads to the bile duct epithelia being exposed to intestinal contents. With regard to the risk factors associated with post-operative intra-hepatic calculi formation, apart from post-operative anastomotic strictures, the other factors include post-operative reflux cholangitis, residual stones, bile stasis and intra-hepatic bile duct expansion. In our study, 8.2% of patients in the PBD 0-cm group and 10.4% in the PBD 1-cm group were alive with post-operative intra-hepatic calculi formation. In the 14 patients of the PBD 0-cm group with post-operative intra-hepatic calculi formation, 10 had reflux cholangitis and 4 had anastomotic stricture. In the 10 patients of the PBD 1-cm group with post-operative intra-hepatic calculi formation, 7 had anastomotic stricture and 3 had reflux cholangitis, which once again confirmed the high correlation between post-operative intra-hepatic calculi formation and anastomotic stricture and reflux cholangitis except surgical procedures. As the incidence of reflux cholangitis (10.5% vs 14.6%) and intra-hepatic calculi formation (8.2% vs 10.4%) was not significantly different between the the PBD 0-cm and 1-cm groups, it seems that leaving behind part of the cyst wall to facilitate hepaticoenterostomy does not reduce the risk of post-operative reflux cholangitis and calculi formation.

The bile duct epithelia are exposed to the intestinal contents mixed with bile and intestinal fluid after reconstruction, which is a risk factor for malignancy. Cancers arising in the remnant cyst walls have been reported to be detected after 2–20 years [9,24,25]. CC associated with biliary malignancy has extremely unfavorable outcomes, with a reported median survival of 6–21 months. The present series showed biliary enteric anastomotic cancer at 10 months in one patient in the PBD 1-cm group and the presence of an unresectable malignant transformation in the intra-pancreatic remnant distal stump at 5 months in one patient in the PBD 1-cm group and at 25 months in one patient in the PBD 0-cm group. Cho *et al.* also reported that cancer developed at the anastomotic site after only 6 and 13 months, suggesting that precancerous changes may have been present but not diagnosed at the time of the initial surgery [12]. We found atypical hyperplasia in the proximal cyst wall when we reviewed the post-operative pathological examination of the biliary enteric anastomotic cancer patients. It is believed that intra-operative frozen biopsy should be routinely performed no matter the proximal or distal cyst walls to exclude canceration of cysts [23]. Our relatively short follow-up period may preclude objective evaluation of the risks of malignant transformation after cyst excision. Based on the findings in the two patients who had a malignant transformation in the remnant distal stump at 5 and 25 months, respectively, it seems that the intra-pancreatic cystic remnant and anomalous pancreaticobiliary duct union might be associated with malignant changes [6,12,26]. To improve outcomes, the intra-pancreatic portion of CC should be resected completely at the time of primary surgery resection of a CC [16].

Our study has several limitations. The first is the retrospective design in which there is a problem of selection bias and the numbers in both arms may not be comparable. Second, our sample size was small, especially in the PBD 1-cm group, and was drawn from a single geographic area. Third, the follow-up period is relatively short. Future work is warranted to estimate the accurate effect of the remnant proximal cyst wall on the outcome.

In conclusion, we reviewed 267 patients diagnosed with type I CC and found that leaving 1 cm of the proximal cyst wall to facilitate hepaticoenterostomy does not reduce the risk of post-operative complications, including cholangitis, anastomotic stricture, reflux cholangitis or calculi formation. Ease of performing anastomosis does not justify retaining a segment of CC due to a higher risk of anastomotic stenosis and leaves the part of the mucosa at risk of developing malignancy. Therefore, a complete excision of the CC performed with anastomosis to the healthy proximal bile duct is necessary in type I CC. However, randomized controlled trial research and long-term observation should be conducted to determine the impact of remnant cyst wall on post-operative morbidity and malignancy in current treatment of adult type I CC.
